# Elucidation of Softening Mechanism in Rinse Cycle Fabric Softeners. Part 1: Effect of Hydrogen Bonding

**DOI:** 10.1007/s11743-015-1732-4

**Published:** 2015-10-05

**Authors:** Takako Igarashi, Naoki Morita, Yoshimasa Okamoto, Koichi Nakamura

**Affiliations:** R&D-Household Products Research, Kao Corporation, 1334 Minato, Wakayama-shi, Wakayama 640-8580 Japan; R&D-Analytical Science Research, Kao Corporation, 1334 Minato, Wakayama-shi, Wakayama 640-8580 Japan

**Keywords:** Fabric softener, Softening mechanism, Cotton, Polyester, Bound water, Hydrogen bonding, Fiber cross-linking

## Abstract

Most softening agents, such as rinse cycle fabric softeners, used by consumers at home contain cationic surfactants that have two long alkyl chains as their main component. The softening mechanism on fibers, especially cotton, has not yet been scientifically established, despite the market prevalence of fabric softeners for decades. One explanation for the softening effect is that the friction between fibers is reduced. According to this explanation, the fiber surfaces are coated by layers of alkyl chains. Because of the low coefficient of friction between alkyl chain layers of low surface energy, the fibers easily slide against one another yielding softer cotton clothing. However, no direct scientific evidence exists to prove the validity of this explanation. The softening mechanism of cotton yarn is discussed in this paper. Bending force values of cotton yarn treated with several concentrations of softener are measured by bend testing, and cotton and polyester yarns are compared. Results indicate that increases in cotton yarn hardness after natural drying are caused by cross-linking among inner fibers aided by bound water. This type of bound water has been known to exist even after 2 days of drying at 25 °C and 60 % relative humidity. Yarn dried in vacuo is soft, similar to that treated with softener. Thus, some of the softening effect caused by fabric softeners on cotton can be attributed to the prevention of cross-linking by bound water between cotton fibers.

## Introduction

**Fig. 1:**
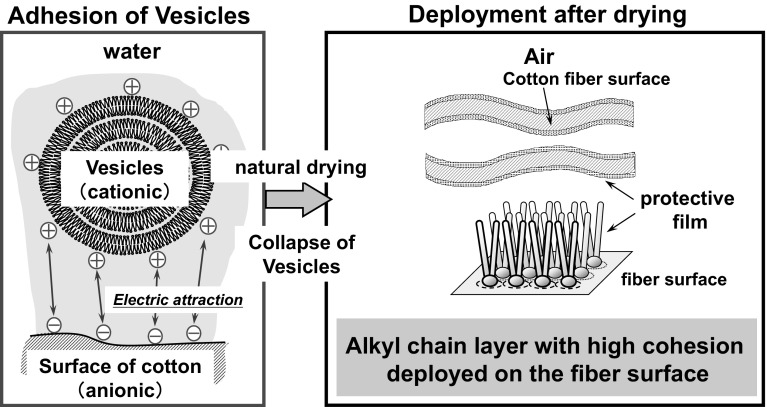
Electrostatic interaction of cationic vesicles with negatively charged fibers (*left*) and collapse of vesicles to cover fiber surface with layers of hydrophobic alkyl chains (*right*)

Most current softening agents used in home fabric softener (hereafter referred to as softener) contain cationic surfactants that have two long alkyl chains as their main component. The current explanation for the softening mechanism is that softener lowers the fiber-to-fiber friction. According to this explanation, cationic vesicles in water are adsorbed to the surfaces of the fibers, which are electrostatically negatively charged, as shown in the Fig. 1. During the drying process, vesicles collapse and the fiber surfaces are covered by layers of the hydrophobic alkyl chains that have low surface energies in air. It is believed that this process causes a reduction in friction between fibers, leaving cotton clothes softened [1–10]. Thus, the softness of clothes after using a softener depends on the friction between fibers.^1^

Since the 1930s, there have been many reports of fabric undergoing stress changes caused by external forces. Softener was introduced to the market in the USA in 1955. After the introduction of softener, Evance [14] reported, in his review, that there were two primary reasons for the softening effect: the plasticizing effect and the friction-reducing properties. For cotton fibers, water is known to be the most effective plasticizing agent. High water content yields high flexibility, and in Evance’s review, polyalcohol and hydroscopic salts, such as calcium chloride, were said to have the same plasticizing effect. However, a change in the strength of a single fiber (the plasticizing effect) could not be detected when cationic surfactants were used. Thus, the softening agent was determined not to penetrate into single fibers, because the vesicles are very large. As a result, researchers attributed the softening effect to lubrication on the surface of the fibers. Some reports interpret the softening effect as a result of frictional changes between cloth and fibers. This fiber surface lubrication theory, however, has not been supported by any direct positive experimental results. Larrat [15] reported experimental results from a friction meter and handle-o-meter. In friction meter testing, a reduction in friction between the fibers was found with increasing softening effect. But the handle-o-meter did not detect a relationship between the touch of softness and the friction reduction. Roeder [16] and Olofsson and Gralen [17, 18] reported that the positioning in between the static and dynamic coefficient of friction plays an important role when we discuss the softness and creaks. To date, their reports have been cited many times and seem to be the basis of current softening theory. The fact that the coefficient of friction correlates with the degree of the softness, however, does not necessarily prove that these properties are dependent on one another, and the fiber surface lubrication theory has not yet been verified by direct experimental results. Motoyama and Saiuchi [19] reported the importance of friction in the softening effect, but they also acknowledged that the absolute friction value and the trend of friction data depend on the measurement methods. It is suggested that a fiber-softening agent must decrease the frictional force between fibers and human skin. However, in another report, Crutzen [11] suggests that the effective softening agent does not necessarily reduce the frictional force.

Recently, Sebastian et al. [20] attempted to evaluate the junction rupture force value (JRF) by drawing one thread from a woven fabric. The JRF value is related to the coefficient of static friction and could be a proper evaluation index of softness, but they also noted that the measured value did not solely result from the frictional force at the junction, as this phenomenon is considered to include not only frictional force but also the adhesive force. Motoyama and Saiuchi [19] also investigated physical fiber properties other than friction, but these were not discussed in the context of softness. Inoue et al. [21] reported that the values (NUMERI, FUKURAMI, SOFUTOSA) measured by Kawabata’s evaluation system (KES) [22] increase when softener is used, and they discuss that properties such as friction force, surface roughness, and elasticity recovery cause these phenomena.

Although there are various theories about the softening mechanism, there has been no sufficient verification to date. If towels are sufficiently washed to remove pretreatment agents with solvents that are normally used in their manufacture, they typically become hard after natural drying. We focus on this hardness and the three-dimensional shape of a naturally dried towel after wetting, and consider that these properties are not caused by friction. These properties are attributed to the phenomenon of “solidification”. Thus, in this study we investigate the softening mechanism of fabric softeners by analyzing the cause of solidification.

## Experimental Section

### Cotton and Polyester Fibers

Softening experiments were performed using both cloth and yarn samples. Cotton towels (TW220, Takei Corp., Japan) and polyester faille were used as clothes. Cotton (20#, Yokota Corp., Japan) and polyester (20#, King Corp., Japan) were used as yarns. Before experiment, these fibers were prewashed by two methods (A and B) to remove any fiber treatment agents used in their manufacture.

In method A, the samples were prewashed using a fully automatic washing machine. Twenty-four cotton towels and 52.22 g of nonionic detergent (Emulgen108, Kao Corp., Japan, 10 % aqueous solution) were loaded into the washing machine (NA-F702P, Panasonic Corp., Japan) with 47 L of water and washed in two steps as follows: (1) the samples were washed for 9 min (with water containing the aforementioned nonionic detergent), rinsed twice with water, and spun dry for 3 min. This step was repeated three times: (2) the samples were washed for 9 min (with water only), rinsed twice with water, and spun dry for 3 min. This step was repeated twice.

In method B, the samples were prewashed with organic solvents. Cotton towels were first cut into pieces (8 cm × 8 cm) and prewashed using method A. Then they were washed again, stirring in 300 mL CHCl_3_/MeOH (1:1 wt. ratio) for 5 min in a beaker. This procedure was repeated five times. For polyester faille and cotton yarns, only solvent washing was applied.

### Softener Treatment Methods

Distearyldimethylammonium chloride (Tokyo Kasei Corp., Japan) was used as a model softener without further purification. The softener treatment methods were chosen from the methods described below. Softener treatment concentrations were set to 0, 0.05, 0.1 (standard concentration), and 0.3 o.w.f. (on the weight of fabric).

Softener treatment of cotton towels using a washing machine: Three cotton towels prepared using method A were treated with an aqueous solution containing the softening agent in a small washing machine (MiniMini Washer NA-35, Panasonic Corp., Japan). In this process, the bath ratio (ratio of water weight to cloth weight) was set to 25, and tap water from Wakayama city, Japan was used at 25 °C. The softening agent was first dispersed in the water, and three towels were added while stirring for 5 min. Previous studies have shown the adsorption of the softener to be nearly 100 % within 5 min for these towels. After treatment with softener, the towels were handled by two different methods depending on the purpose: (1) dried naturally from the wet condition, or (2) dried naturally after spin drying with a two-tank washing machine for 3 min (PS-H35L, Hitachi Corp.) and fluttered (five times by hand).

Softener treatment of cloth using a stirrer: Three cloth pieces (8 cm × 8 cm) prepared by method B were put into 300 mL of ion-exchanged water in a 500-mL beaker. The contents of the beaker were stirred for 5 min, and a predetermined amount of softener (0.5 % aqueous dispersion) was added, followed by stirring for another 5 min. The cloths treated with softener were spread and dried naturally on a sheet of polypropylene (PC-8186 produced by Sekisuikagaku Corp., Japan, hereafter referred to as PP) which were prewashed with dishwashing detergent.

**Fig. 2:**
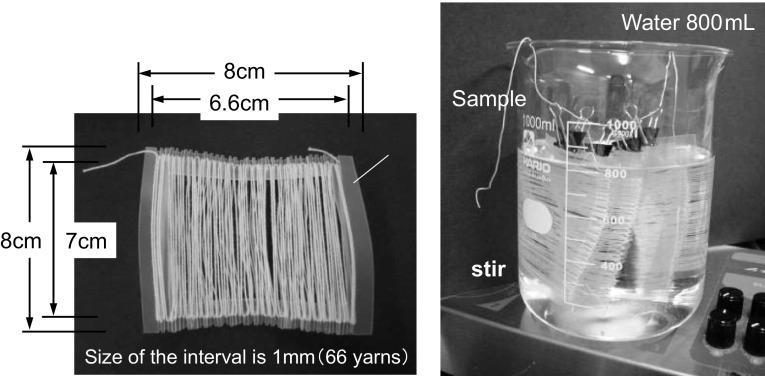
Softener treatment of cotton yarns. The cotton yarns prepared by method B were fixed on a PP frame as shown on the *left*. The yarn was immersed in deionized water and stirred for 5 min, after which the softener was added and stirred for 120 min as shown on the *right*

Softener treatment of cotton yarns using a stirrer: the cotton yarns (ca. 2 g) prepared by method B were fixed on a PP frame as shown on the left side of Fig. 2. The yarn was immersed in 800 mL of deionized water in a 1000-mL beaker and stirred for 5 min, after which the softener (0.5 % aqueous dispersion) was added and stirred for 120 min. The resulting treated yarn was dried naturally.

Softener treatment of polyester cloth using a washing machine: polyester cloth (200 g) prepared by method A was treated with an aqueous solution containing softening agent in a small washing machine (MiniMini Washer NA-35, Panasonic Corp., Japan). For this process, the bath ratio was set to 25, and tap water from Wakayama city, Japan was used at 25 °C. First, a sufficient amount of softening agent was dispersed in the water, followed by the immersion of the polyester cloth in the tap water while stirring for 5 min. After treatment with softener, the cloth was spin-dried with a two-tank washing machine for 3 min (PS-H35L, Hitachi Corp.), fluttered (five times by hand), and dried naturally.

### Measuring Adsorption of Softening Agent

Yarns (ca. 1.0 g) prepared by the method described above, 80 g of methanol (HPLC grade, Wako Pure Chemical Industries Ltd., Japan), and 0.8 g concentrated HCl (35 % aqueous solution, Wako Pure Chemical Industries Ltd., Japan) were poured into a 100-mL glass container and treated with ultrasonic agitation for 20 min. The resulting liquid was diluted by 100–1000-fold with methanol. Samples were measured three times by liquid chromatography–mass spectrometry.

### Equipment and Measurement Conditions

High-performance liquid chromatography–mass spectrometry (HPLC–MS) was used to measure the amount of the softening agent adsorbed onto the clothes and yarns. The equipment used for this study was an HPLC–MS system (Prominance UFLC, Shimazu Seisakusyo, Japan) and mass spectrometer (LCMS-2010, Shimazu Seisakusyo, Japan) with electrospray ionization (ESI) performed in the positive mode. Ionization conditions for electrospraying were optimized by the automatic calibration system. An analytical column, Unison UK-C18 HT (diameter 2 mm × 50 mm, 3 μm, Imtakt, Japan) was operated at 40 °C. Mobile phase A comprised 10 mM aqueous ammonium acetate solution. Mobile phase B comprised 10 mM ammonium acetate solution in methanol. The gradient condition was set from 50 to 100 % phase B over 2 min and maintained for 3 min at a flow rate of 0.5 mL/min. For selected ion monitoring measurements, each of the protonated molecular ions [M+H^+^] was used:* m*/*z* 550.7 for distearyldimethylammonium chloride.

### Cloth and Yarn Hardness Measurements

**Fig. 3:**
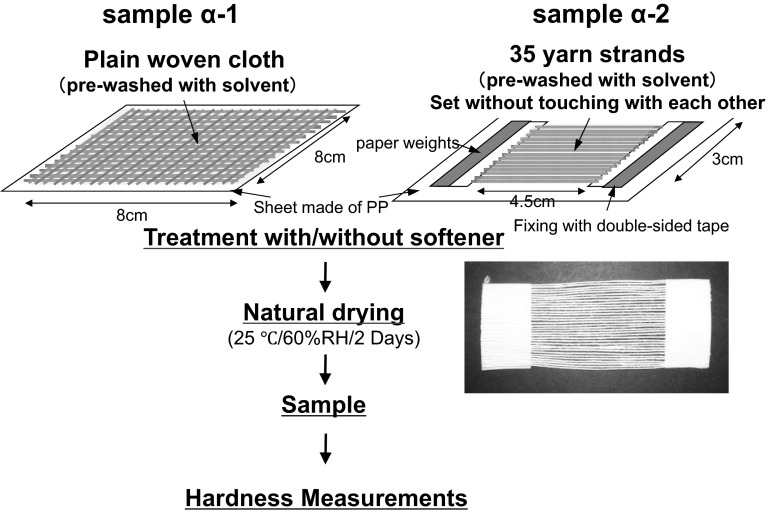
Process for preparing cloth and yarn samples for hardness measurements. Cloth samples were spread on a sheet of polypropylene, soaked in deionized water, and left to dry at 25 °C and 50 % RH for 2 days (naturally dried). Yarn samples were fixed parallel to one another with double-sided tape so that they do not touch, soaked in deionized water, and naturally dried

Cloth and yarn samples were prepared using the method described in Fig. 3. Hereafter, cloth and yarn samples made of cotton are named α-1 and α-2, and cloth and yarn samples made from polyester are named β-1 and β-2, respectively. Cloth samples, α-1 and β-1, were prepared by the method described above. Cloth samples were spread on a sheet of polypropylene, soaked in deionized water, and left to dry at 25 °C and 50 % RH for 2 days (naturally dried). Yarn samples, α-2 and β-2, were prepared by the method described previously. Thirty-five lengths of yarn were fixed parallel to one another with double-sided tape on graph paper (4.5 cm × 3 cm) so that they do not touch each other. These samples were set on the polypropylene sheet, soaked in deionized water, and naturally dried.

### Pure Bending Test

**Fig. 4:**
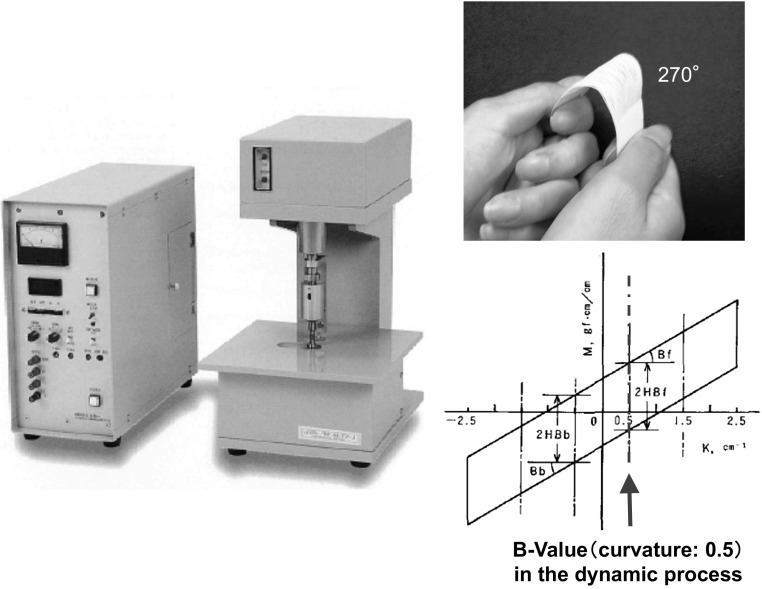
Automatic pure bending tester employed to measure bending force

An automatic pure bending tester (KES-FB2-AUTO-A, Kato Tech Corp., Japan) (Fig. 4) was employed to measure each sample. Cloth and yarn samples prepared by the methods described previously were loaded into the tester chucks (8 cm × 1 cm) and bent to 270° with a maximum curvature factor of ±2.5 cm at a deformation rate of 0.5 cm/s. The *B* values (gf cm^2^/cm) were measured thrice at a curvature factor of 0.5 cm during the dynamic process at 25 °C and 50 % RH.

### Water Detection by Near-Infrared (NIR) Measurements

An Infra SpecNR800 (Yokokawa Denki Corp., Japan) was used at 25 °C and 50 % RH to detect the quantity of water present in each sample. Measurement was made over the wavelength range of 4000–9000 cm^−1^ for 1024 scans with a resolution of 8 cm^−1^.

### Removal of Bound Water in Cotton by Complete Drying

**Fig. 5:**
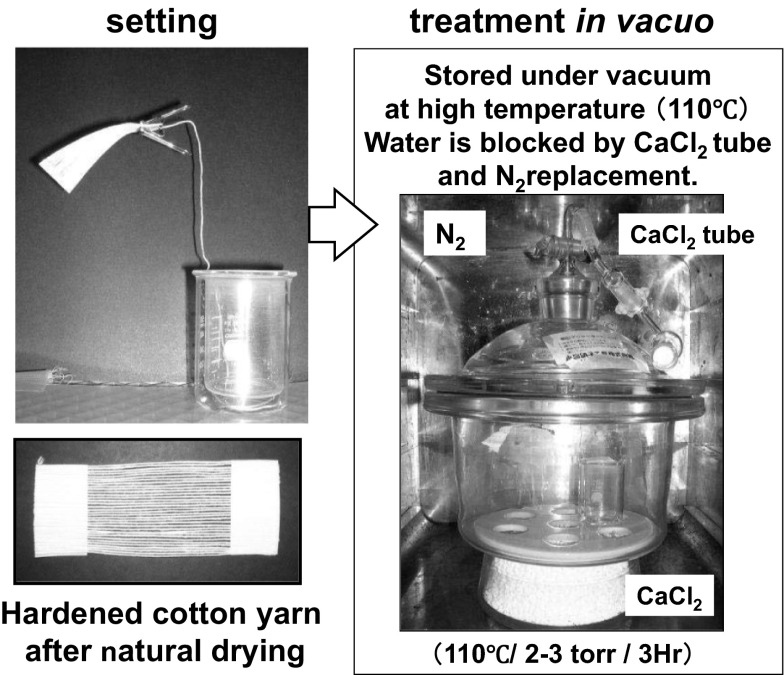
Removal of bound water in cotton by complete drying in a vacuum desiccator equipped with a calcium chloride tube (ca. 2–35 torr) at 110 °C for 3 h

A sample made of cotton yarn prepared by the methods described previously was fixed as shown in Fig. 5. The sample was put into a desiccator equipped with a calcium chloride tube and kept under vacuum (ca. 2–35 torr) at 110 °C for 3 h. In the final stage, pressure was released under dry N_2_ gas conditions to prevent water from contaminating the sample. This measurement was done to three separate samples.

### Evaluation of Softening Effect

The degree of softness was evaluated by sensory tests performed by five expert panelists.

## Results and Discussion

### Influence of Treatment Conditions on Softness

**Fig. 6:**
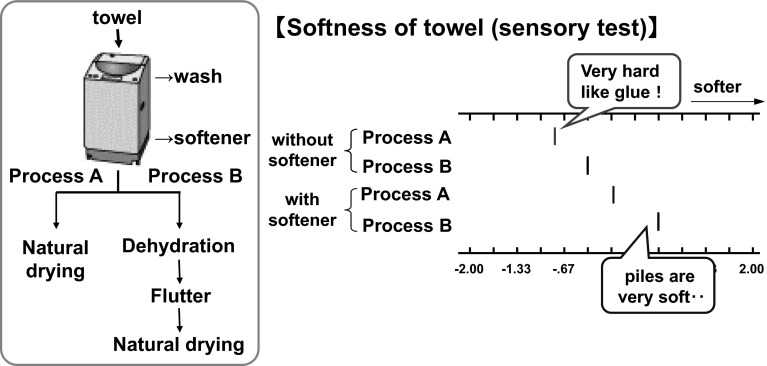
Influence of treatment conditions on softness. Process A corresponds to natural drying after washing with water. Process B corresponds to natural drying after washing followed by additional dehydration and fluttering

Differences in softness using the treatments described in Fig. 6 were observed. Process A corresponds to natural drying after washing with water. Process B corresponds to natural drying after washing followed by additional dehydration and fluttering. We prepared towels using both processes, with and without softener, and evaluated the appearance and softness. For towels treated with process A without using softener, a change in appearance was recognized, i.e., fluff and pile on the surfaces of each towel were compressed. However, when the towels were treated with process B, the pile was more irregular and single fibers were fluffed. When comparing the softness, the towels treated with process A were harder. This result suggests some binding or linking exists between fibers.

**Fig. 7:**
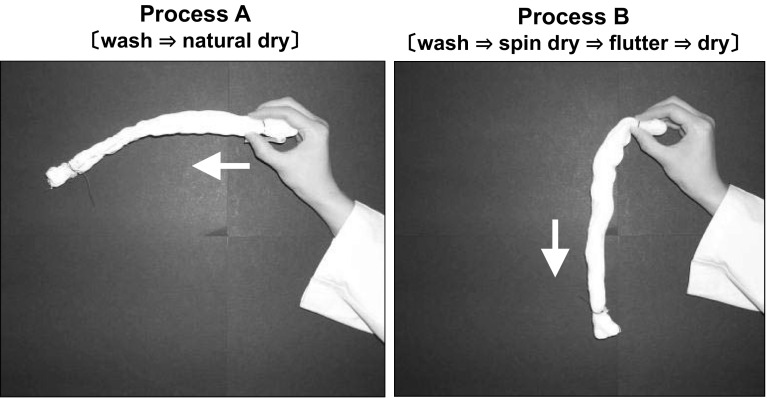
Influence of treatment conditions on stiffness of cotton yarn. Process A corresponds to natural drying after washing with water. Process B corresponds to natural drying after washing followed by additional dehydration and fluttering

When softener (0.1 % o.w.f.) was used, the softness significantly improved. In comparing processes A and B, towels treated with process B had more volume and higher softening effect. Verification of this softening phenomenon was attempted by studying cotton yarn bundles (pretreatment agent already removed). The results show a difference in the appearance and softening effects between processes A and B. Process A caused the cotton yarn bundle to be compressed, as the diameter of the yarn bundle decreased (the distance between the fibers was also reduced). The bundle shape was maintained horizontally without sagging under gravity, as shown in Fig. 7 (left). Sensory tests showed that yarn bundles treated with process A were apparently harder than those treated with process B. These phenomena demonstrate the existence of a hardening factor when the yarns bundles did not contain any fiber treatment agent.

On the basis of the knowledge that the secondary wall of a cotton fiber is primarily made out of cellulose and the surface of the cotton fiber is covered by hydroxyl groups, it is reasonable to consider that the main factor in causing this type of hardness is hydrogen bonding. This bonding force binds and links cellulose fibers to one another, imparting hardness to the cotton yarns and cloth.

### Analysis of Binding Force Among Single Fibers

**Fig. 8:**
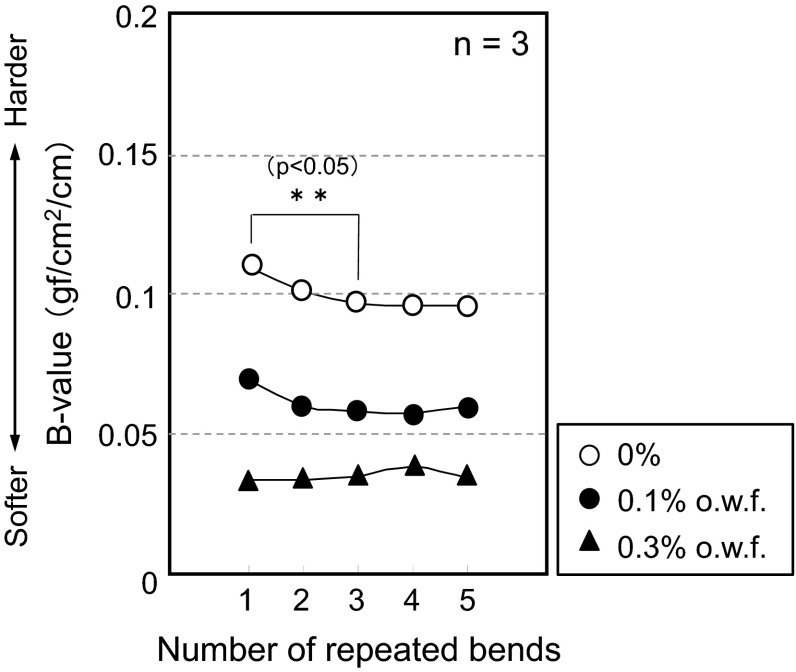
Cotton cloth bending force values. The *horizontal axis* represents the number of bending cycles and the *vertical axis* represents the moment of bending [average of the *B* value (*n* = 3)]

To interpret the hydrogen bonding phenomenon, cloth bending force measurements were used to quantitatively estimate the amount of hydrogen bonding. The concentration of softener for α-1 was varied from 0 to 0.3 % (0.1 % is the standard concentration of fabric softener), and the bending properties were measured. In Fig. 8, the horizontal axis represents the number of bending cycles and the vertical axis represents the moment of bending [average of the *B* value (*n* = 3)]. In this experiment, the *B* values were measured in five consecutive bends of three samples prepared under the same conditions. The *B* value decreased for samples treated with softener, as expected, and approached a constant value above concentrations of 0.1 %. This reduction in hardness (and increase in softness) is caused by the softener and is the phenomenon that is encountered in daily life. Interestingly, a reduction in bending force was observed between the first and second bends during the testing procedure (risk rate *p* < 0.05). However, no further reductions were apparent after the third bending.

**Fig. 9:**
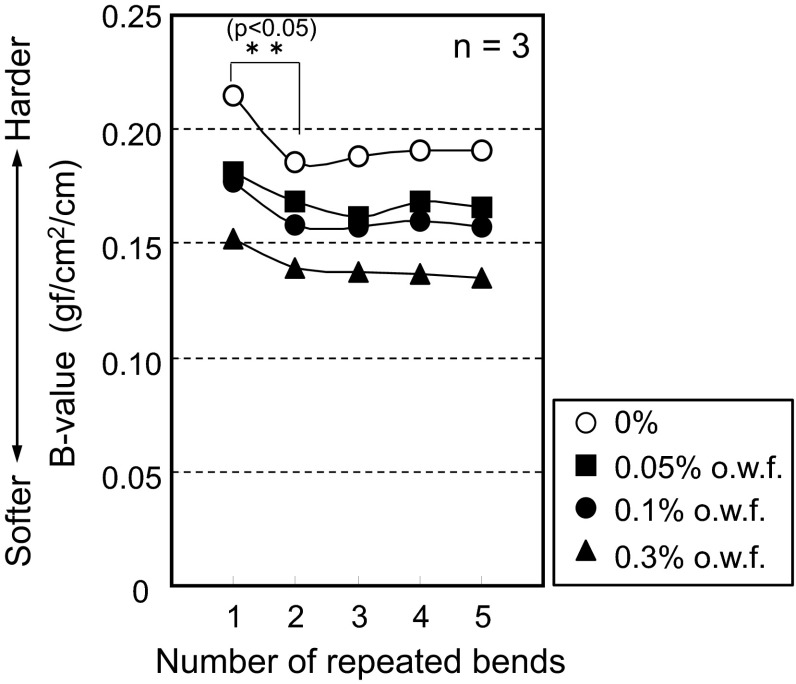
Cotton yarn bending force values. The *horizontal axis* represents the number of bending cycles and the *vertical axis* represents the moment of bending [average of the *B* value (*n* = 3)]

To identify whether hydrogen bonding is occurring within or on the surface of the yarn, similar bending force measurements using α-2 were carried out. The adsorption weights of the softening agent on the sample were 0 mg/kg (0 %), 353 mg/kg (0.05 %), 1050 mg/kg (0.1 %), and 1100 mg/kg (0.3 %), respectively. Longer treatment times (48 min) were needed for yarn because an adsorption percentage (30 %) was found in the case of a 5-min treatment. Normally, 5 min is enough time for cotton cloth to show 100 % adsorption. This low adsorption percentage is likely because the yarn moves in a synchronized manner with water flow while stirring. The chances of collision between yarns and softener vesicles apparently decrease. Thus, longer times are required to achieve 100 % adsorption percentage. A reduction in yarn hardness was observed with increasing concentration of softening agents, and a decrease in hardness upon initial bending was also observed (risk rate *p* < 0.05) (Fig. 9). The softening effect is more apparent in yarn.

We suggest that the decrease in hardness is explained by the prevention of hydrogen bonding between fibers. When the concentration of softening agent was set to 0.3 % o.w.f., there was no longer a recognizable change in the *B* value over the course of repeated bend tests. This phenomenon implies that the most effective inhibition of hydrogen bonding between fibers occurred at the 0.3 % o.w.f. softener concentration.

**Fig. 10:**
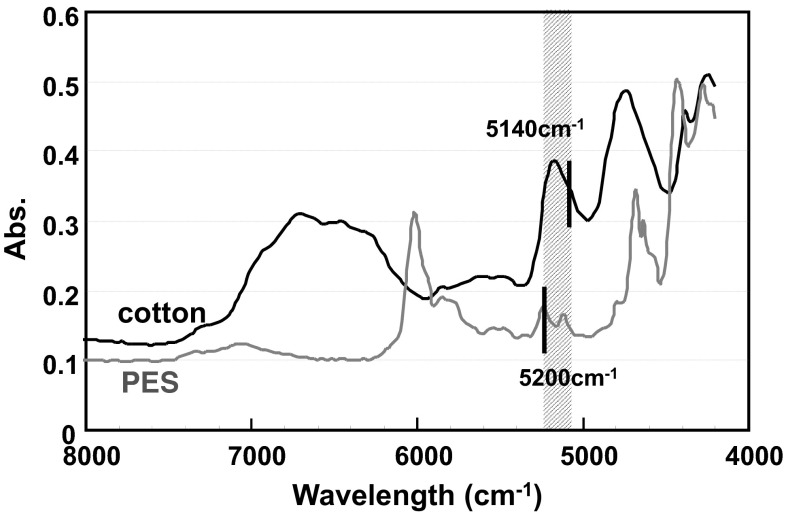
Comparison of water content from the NIR spectra after environmental conditioning (25 °C, 50 % RH)

To verify the hydrogen bonding results in cotton, the bending properties of polyester cloth was measured. The official value of moisture content reported for polyester is 0.4 wt% (25 °C and 50 % RH) [12], which is considerably less than that of cotton (8.5 wt%) [23]. Comparing water contents from the NIR spectra (Fig. 10) after environmental conditioning (25 °C, 50 % RH), the absorbance intensity of bound water (wavelength is 5144 cm^−1^) in polyester is significantly reduced compared to cotton. Therefore, we can consider polyester to have less water and hydrogen bonding is not abundant.

**Fig. 11:**
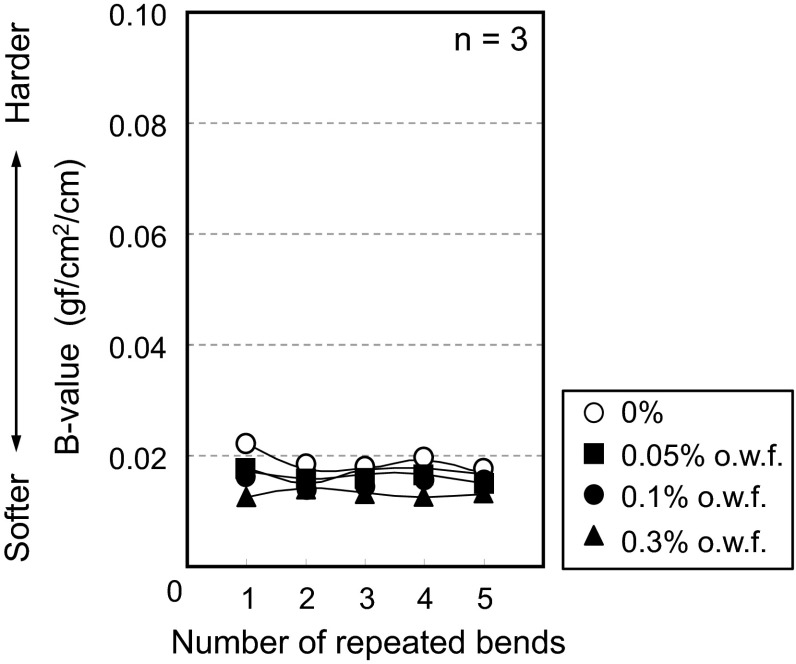
Polyester cloth bending force values. The *horizontal axis* represents the number of bending cycles and the *vertical axis* represents the moment of bending [average of the *B* value (*n* = 3)]

**Fig. 12:**
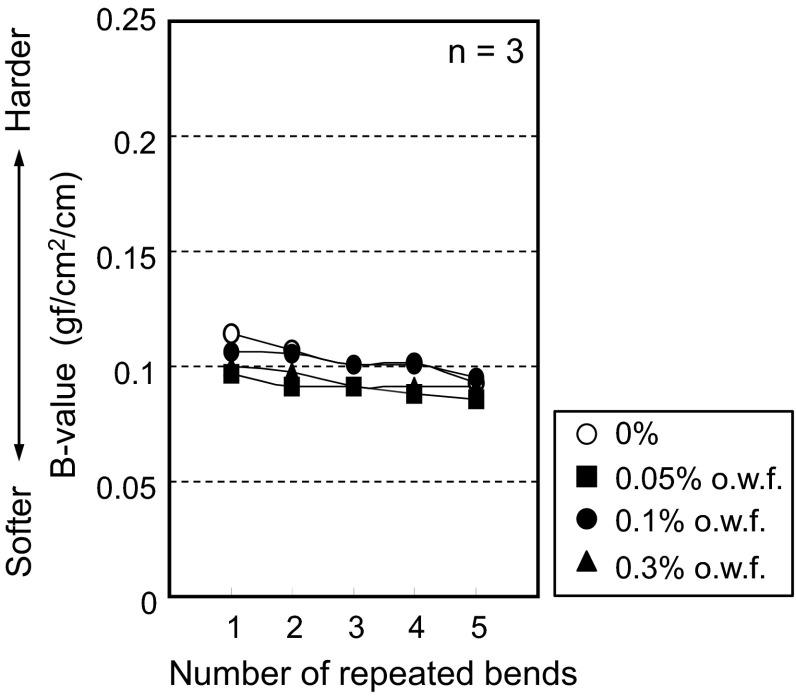
Polyester yarn bending force values. The *horizontal axis* represents the number of bending cycles and the *vertical axis* represents the moment of bending [average of the *B* value (*n* = 3)]

Automatic pure bending tests were carried out on polyester faille using the same methods for cotton. Results in Figs. 11 and 12 show that a change in *B* value is not observed throughout repeated bending tests for both cloth and yarn respectively. These results strongly suggest that the initial reduction in the *B* value for cotton is caused by hydrogen bonding.

### Possible Softening Mechanisms by Fabric Softener

On the basis of the results described previously, the softening mechanism is interpreted as follows. First, when cotton cloth or yarn is dried naturally, an increase in hardness is observed. This hardness is caused by the cross-linking of cotton fibers by hydrogen bonds. During the natural drying process from the wet condition, a meniscus exists between single fibers, and the influence of surface tension increases as water is removed. Campbell [24] reported that if the distance between fibers is as close as 1 nm, attraction forces as high as 1.5 t/cm^2^ can be reached. Kohata et al. [25] reported that six layers of bound water exists on the surface of cotton fiber after natural drying on the basis of simulations of surface area obtained by Brunauer–Emmett–Teller adsorption theory. Bound water (non-frozen) is not liquid, but is rather solid like ice at room temperature, and bound water functions as a cross-linking agent between fibers, imparting hardness in dried cotton. Van den Akker [26] reported when water in wet cellulose paper was sublimated, the paper displayed a bulky appearance. This bulkiness was likely caused by the absence of a meniscus force during water vaporization. Additionally, when cotton fibers are dried naturally after being wet with CHCl_3_, which generates no meniscus force, the bulkiness of the fabric also increases.

When a bending force is applied to hardened cotton yarn that is cross-linked by hydrogen bonding, the hydrogen-bonding network collapses and the hardness is reduced. This conclusion is based on the change that occurs when the clothes are flattened and/or kneaded.

**Fig. 13:**
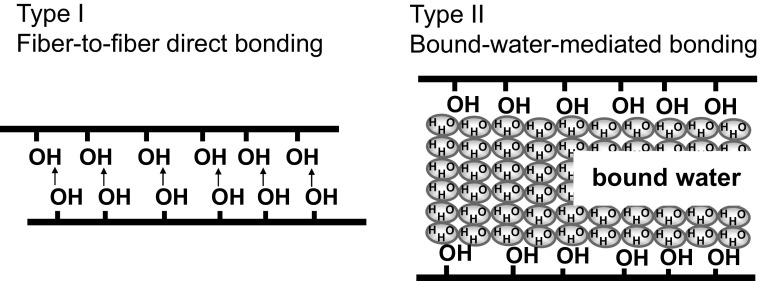
Types of hydrogen bonding structures may exist in the naturally dried cotton yarns. Type I is characterized by direct hydrogen bonding among OH groups on the surfaces of fibers. Type II consists of hydrogen bonding between the fibers that have water bound on their surfaces

**Fig. 14:**
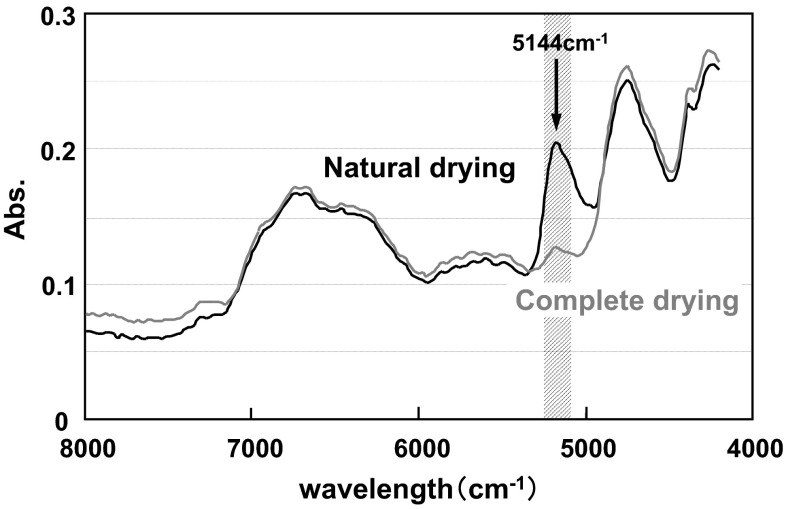
Comparison of NIR spectra between natural and complete drying. The absorbance intensity of water at 5144 cm^−1^ had significantly decreased, signifying that nearly all bound water was removed

**Fig. 15:**
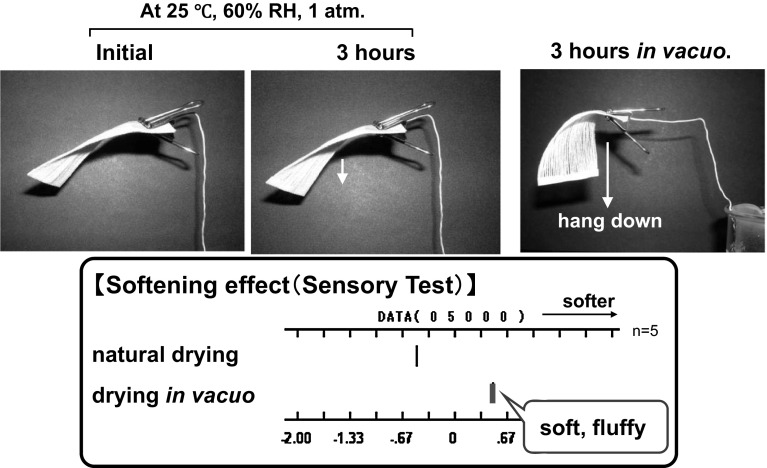
Appearance and texture of cotton yarn after complete drying

In the theory mentioned above, two types of hydrogen bonding structures may exist in the naturally dried cotton yarns (Fig. 13). Type I is characterized by direct hydrogen bonding among OH groups on the surfaces of fibers. Type II is hydrogen bonding between the fibers that have water bound on their surfaces. To identify which type of hydrogen bonding is predominant, yarn samples were completely dried in vacuo at 110 °C and ca. 2 torr for 3 h. Cotton does not have a melting temperature or a glass transition temperature and decomposes at approximately 240 °C. When the NIR spectra are compared after complete drying, the absorbance intensity of water at 5144 cm^−1^ had significantly decreased, signifying that nearly all bound water was removed as shown in Fig. 14. After complete drying, the appearance and texture of the yarn sample had changed, as the yarn hung in the desiccator (Fig. 15) and became softer. These results suggest that the hardness introduced by natural drying had reduced. A lot of fluff on the surface layer was also observed after complete drying. The final state of the yarn was quite similar to that of yarn treated with fabric softener.

On the basis of the results and observations mentioned above, the additional hardness that was introduced by natural drying is primarily caused by a hydrogen-bonding network that includes bound water between single fibers (type II). The increase in hardness after complete drying was not observed when the samples were stored in a dry atmosphere, such as dry nitrogen gas. On the basis of this interpretation, the softening mechanism of fabric softeners is mainly attributed to the prevention of a cross-linked network by bound water.

## Conclusion

The hardening phenomenon that cotton yarn experiences after natural drying is reportedly caused by the formation of a cross-linked network involving hydrogen-bonded water as an intermediary. In addition to the hardening, the distances between the single fibers in cotton are shortened by the meniscus force during drying. On the basis of this explanation and the observation that the absolutely dry sample is very close to that of the softener-treated yarn, the softening effect of fabric softener in cotton is understood mainly to be caused by the prevention of hydrogen bonding in the presence of bound water. We propose that the softening mechanism in fabric softener is not considered only to be caused by a decrease in frictional force between fibers, but the prevention of hydrogen bonding by the formation of a hydrophobic layer of softener molecules is also important.

## Footnotes

1 Since the softening agent exists as a vesicle with a cationic charge in water, this cationic vesicle is considered to be adsorbed to the surfaces of the cotton fibers, which are electrostatically negatively charged. Cotton surfaces are negatively charged because cotton consists of cellulose that contains anionic carboxyl groups that are generated by oxidation of OH groups. On the other hand, Crutzen [11] suggests that hydrophobic interactions derived from the long alkyl chains of cationic surfactants are the primary driving force for adsorption, because the softening agent can adsorb onto fiber surfaces that are not charged. Minegishi and Arai [12] report that the time required for the softening agent to reach adsorption equilibrium is strongly dependent on mechanical force. This result suggests that the driving force for adsorption is dominated by the frequency of collisions between fibers and vesicles. Okumura and Yokoi [2] and Nakamura et al. [13] have also reported on the adsorption state of the softening agent on fibers; however, the various theories are still not well understood.
